# Discrimination of spectral reflectance under environmental illumination

**DOI:** 10.1364/JOSAA.35.00B244

**Published:** 2018-03-12

**Authors:** Takuma Morimoto, Hannah E. Smithson

**Affiliations:** Department of Experimental Psychology, University of Oxford, 15 Parks Rd, Oxford OX1 3PH, UK

**Keywords:** (330.1720) Color vision, (330.5510) Psychophysics, (330.6180) Spectral discrimination, (330.1715) Color, rendering and metamerism

## Abstract

Color constancy is the ability to recover a stable perceptual estimate of surface reflectance, regardless of the lighting environment. However, we know little about how observers make judgments of the surface color of glossy objects, particularly in complex lighting environments that introduce complex spatial patterns of chromatic variation across an object’s surface. To address this question, we measured thresholds for reflectance discrimination using computer-rendered stimuli under environmental illumination. In Experiment 1, we found that glossiness and shape had small effects on discrimination thresholds. Importantly, discrimination ellipses extended along the direction in which the chromaticities in the environmental illumination spread. In Experiment 2, we also found that the observers’ abilities to judge surface colors were worse in lighting environments with an atypical chromatic distribution.

## 1. INTRODUCTION

Color constancy is the visual ability that allows us to judge the surface colors of objects under various lighting environments. To achieve color constancy, the visual system needs to cancel the influence of illumination and retrieve an estimate of surface color. However, such a separation is an under-constrained problem because we typically have access to only the cone signals elicited by the illumination once it has been spectrally modified by surface spectral reflectance. Considerable past research has been conducted to identify mechanisms of color constancy, as recently summarized by Foster [1], but it is not yet fully understood.

One of the limitations of past studies is the use of simplified stimuli where objects were typically two-dimensional (2D), matte and uniformly illuminated by a single light source [2,3]. Although such stimuli allow careful experimental control, they lack some of the important features of surfaces and illuminants that potentially offer cues for color constancy. One important property of objects in this regard is their specularity. The matte objects that have been used extensively in past studies exhibit only a diffuse reflection whose spectral content is the product of the spectral energy distribution of the illumination and the surface spectral reflectance of the object. By contrast, glossy objects additionally exhibit a specular reflection that carries direct information about the illuminant spectrum, and can therefore provide information about the lights incident on the object’s surface. Thus, color constancy for glossy objects may be supported by some additional mechanisms that are not available for matte stimuli.

Over the past two decades, color vision researchers have attempted to address these questions by utilizing more realistic objects in three-dimensional (3D) setups. Moreover, recent advances in computer graphics techniques have enabled substantial numbers of experiments to investigate various features of material perception [4–6]. In addition to studies on gloss and lightness perception [7–17], there is an increasing number of studies on color perception [18–26].

Nevertheless, despite the significant methodological advances, even these more recent studies have typically ignored the fact that objects in the natural world are not simply illuminated by a single light source, but also receive light that has been reflected from other objects that coexist in the scene. Thus, different locations on an object’s surface receive spectrally different lights from each direction. Consequently, in the proximal image, there is spatial chromatic variation all over the object’s surface. Some regions contain more information about the illumination, whereas others may be dominated by the diffuse component that gives information about surface color. Estimates of surface reflectance, therefore, potentially require a local illuminant discounting mechanism. A handful of studies have considered the influence of multiple illumination regions [27,28], or multiple illuminations that differ in spectral composition and geometry [29,30], or even the effect of spatial variation across the surface of a glossy object that derives from scene-dependent lighting effects [31]. However, the effect of complex environmental illumination that causes abrupt changes in the light reflected from position to position on an object’s surface is still under-explored.

**Fig. 1.**
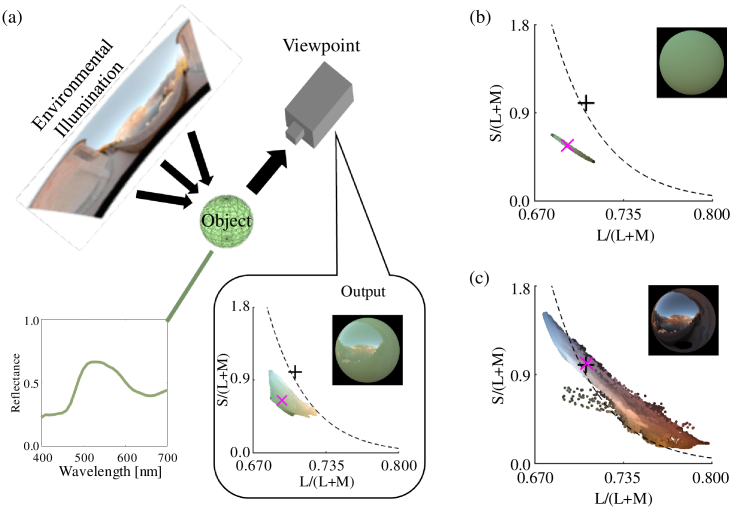
Rendering objects in natural lighting environments. (a) Schematic illustration of the rendering process using environmental illumination. The renderer traces light from the environmental illumination to an object with a particular reflectance, and from there to the viewpoint. The subpanel to the bottom right shows the resultant image of the object and the chromatic distribution of pixels in that image. (b) Chromatic distribution of the diffuse component. (c) Chromatic distribution of the specular component. [(a)–(c)] Chromatic distributions are represented in the MB chromaticity diagram [33]. The black plus symbols indicate equal energy white. The magenta cross symbols indicate the mean chromaticity. The black dashed line indicates the black-body locus.

Experimental study of complex environmental illumination is now possible using a computer graphics technique that stores for a particular point in a scene the incident light from every direction in the environment [32]. Such environmental illumination typically varies in spectral composition from one direction to another. For example, some directions may contain direct sunlight, whereas others are dominated by skylight, or by light reflected from other surfaces in the scene. Importantly, environmental illumination includes not only light emitted directly from a light source, but also mutual reflections from surfaces that coexist in the environment. When we render a test object at the target location within the environment, the technique allows us to simulate the effect of all incident light that hits the object’s surface. For computer graphics, environmental illumination maps are typically stored as an unwrapped 2D image in which each pixel contains incident light from a specific direction. Figure 1(a)

shows an example of such a map and the other components of the rendering process, along with the resultant chromatic distribution of pixels that comprise the rendered glossy object. Unlike a flat, matte surface under uniform illumination, the image of the surface includes a wide range of chromaticities.

As mentioned earlier, chromaticities in the image of the object arise from a combination of two different reflections. Light from any point on the object’s surface can be expressed as a linear weighted sum of the diffuse component and the specular component. Thus, at each point, chromaticity falls between the chromaticity of the diffuse component and that of the specular component. Figures 1(b) and 1(c) shows the chromatic distributions of the diffuse and specular components, respectively. A matte object exhibits only diffuse reflection [Fig. 1(b)], and although chromaticity varies across the surface, the variance is not large. Conversely, the specular component [Fig. 1(c)] reflects the chromaticity distribution of the surrounding environment, which spreads widely, mainly along the black-body locus in this case. For comparison, the color distribution of a glossy object with a single spectral reflectance viewed under simpler illumination such as a single point light source is discussed in detail elsewhere [20].

Environmental illumination is complex in that it introduces spatial chromatic variation on an object’s surface. Some studies have investigated lightness or gloss perception and estimation of lighting direction under environmental illuminations [34–36]. Fleming *et al.* [37] tested the ability to estimate specularity (from matte to glossy) and roughness (from crisp to blurred highlights), and showed that humans judge these properties well, as long as the spatial structure of the specular reflection is representative of the real world. With regard to color vision, Doerschner *et al.* [38] investigated the ability to judge the surface color of a matte surface using the method of achromatic setting, and found that the visual system can eliminate the influence of environmental illumination from a matte surface. However, we still know relatively little about our ability to perceive the surface color of glossy objects under environmental illumination.

The present study specifically aimed to evaluate our ability to discriminate surface colors (surface spectral reflectance) of objects with various properties under complex lighting environments. Such reflectance discrimination could be considered an extension of color discrimination in the presence of chromatic noise [39] because a glossy object could be represented as the diffuse component, which contains the reflectance information, masked by the specular component. The reflectance discrimination task also connects to the literature on illuminant discrimination [40,41] and discrimination of natural objects with variegated surface reflectance [42]. With objects of spatially uniform surface spectral reflectance, such as the objects that we use here, one potential strategy would be to use the mean color across the whole surface. Alternatively, performance may be supported by more specific mechanisms that separate the color that stems from the surface spectral reflectance from the widely spread chromaticities carried in the specular component.

Experiment 1 aimed to explore potential factors that could influence our ability to discriminate the surface spectral reflectance of matte and glossy objects (either spheres or bumpy spheres) under complex environmental illumination. Experiment 2 was designed specifically to investigate whether our visual system exploited the statistical chromatic regularity in the natural world when making a judgment about an object’s color. In both experiments, we used carefully controlled computer-generated stimuli and employed a reflectance-discrimination procedure to identify how much reflectance change was needed to successfully select a stimulus with a different spectral reflectance.

## 2. METHODS

### A. Apparatus

All experiments were computer-controlled and conducted in a dark room. Stimuli were presented on a cathode ray tube (CRT) monitor (NEC, FP2141SB, 21 inches, 1600×1200 pixels) controlled with ViSaGe MkII (Cambridge Research Systems), which allows 14-bit intensity resolution for each phosphor. Gamma correction was performed with a ColorCAL MkII colorimeter (Cambridge Research Systems) and spectral calibration was performed with a SpectroCAL MkII spectroradiometer (Cambridge Research Systems). Viewing distance was maintained with a chin rest positioned 92 cm from the CRT monitor. Observers were asked to view the stimuli binocularly.

### B. Stimuli

#### 1. Rendering

All stimuli were generated by computer graphics techniques. The geometry of each scene (locations of the viewpoint or the camera, an object and an illumination map) was defined using the 3D modeling software Blender (Blender Foundation). Then, rendering was conducted using the physically-based renderer Mitsuba. The resulting multispectral images (31 channels, from 400 nm to 700 nm with 10 nm steps) of the rendered objects were converted to LMS cone coordinates based on Stockman & Sharpe 2° cone fundamentals [43] and then finally converted to RGB values for display on the calibrated CRT. We used Rendertoolbox [44] to automate the production of the multispectral images and MATLAB (MathWorks) to convert the images to RGB images.

#### 2. Environmental Illumination

**Fig. 2.**
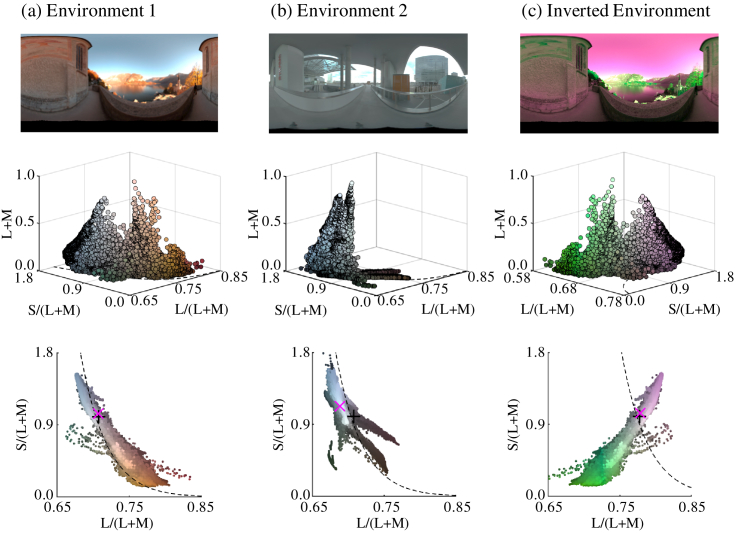
Chromatic properties of the three lighting environments used in the experiments. (a) Environmental Illumination 1 [“Distant Evening Sun (Hallstatt)”], used in Experiments 1 and 2. (b) Environmental Illumination 2 (“Overcast day at Techgate Donaucity”), used in Experiment 1. (c) Chromatically inverted Environmental Illumination 1, used in Experiment 2. [(a)–(c)] The top panel shows a 2D projected image of the 3D environment map. The middle and bottom panels show, respectively, the 3D and 2D color distributions of the environmental illuminations. The magenta cross and black plus symbols indicate, respectively, the mean chromaticity of the distribution and the chromaticity of equal energy white. The black dashed line indicates the black-body locus.

We used two environmental illuminations from a publically available database [45], namely, “Distant Evening Sun (Hallstatt)” and “Overcast day at Techgate Donaucity.” Figure 2

shows images of the environmental illumination maps (top row) and the color distribution of all pixels. Environments 1(a) and 2(b) were used for Experiment 1, whereas Environment 1(a) and its chromatic inversion (c) were used for Experiment 2. We see that chromaticity is distributed along the black-body locus for Environments 1 and 2. These environments were selected to have different mean chromaticities; the mean of Environment 1 is close to equal energy white, whereas the mean of Environment 2 is displaced from equal energy white toward blue-green. In Experiment 2, we used a chromatically inverted environmental illumination to test the effect of the direction of chromatic variation. More detailed rationale is provided in the introduction to Experiment 2. The environmental illuminations were originally 1024×512 images with three channels (RGB), but were promoted to multispectral images within the rendering process by Mitsuba using a method by Smits [46].

#### 3. Object Shape and Specularity

In Experiment 1, two types of object shape were tested (sphere and bumpy), whereas in Experiment 2, we used only bumpy objects. The different object shapes modified the spatial pattern of specular reflection on the object’s surface. The sphere provided a spatially clear reflection of the surrounding environment, whereas the bumpy object provided a distorted reflection. We used two levels of specularity for both experiments: a completely matte surface (control) and a glossy surface with a specular reflectance of 0.20 across all wavelengths as defined within the Mitsuba renderer, which was set to use the Ward reflectance model [47].

#### 4. Surface Spectral Reflectance

**Fig. 3.**
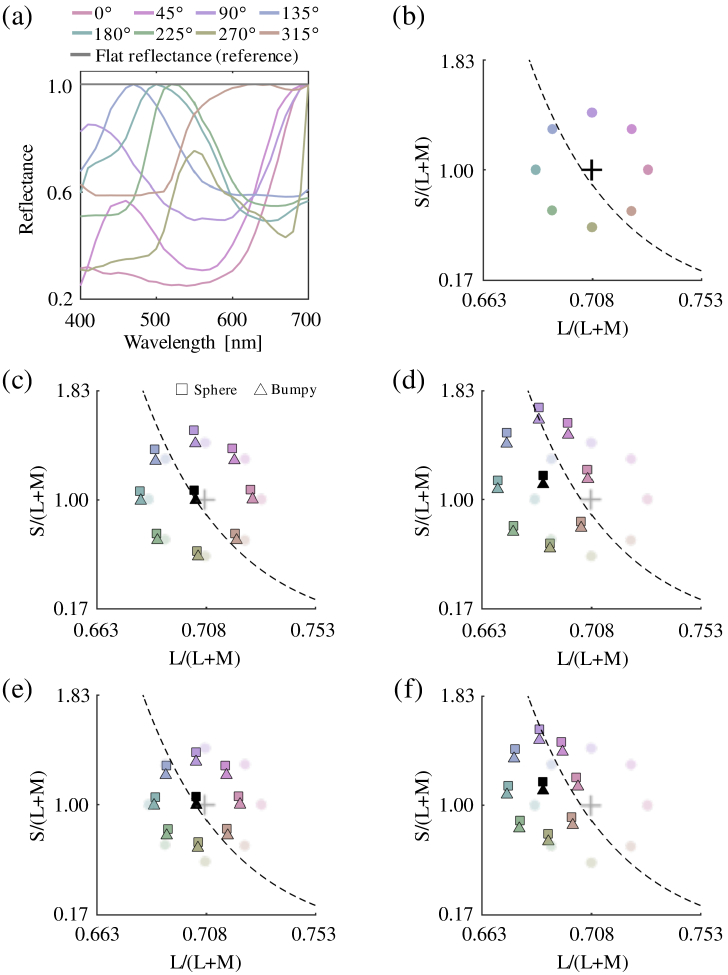
Chromatic properties of the surface spectral reflectance functions used in the experiments. (a) Eight surface reflectances used to measure reflectance discrimination thresholds from the spectrally flat reference reflectance. Each reflectance is independently normalized by its maximum value for the sake of visibility. Note that in the actual experiments they were normalized so that all reflectance functions would produce stimuli of equal luminance when rendered under equal energy white. (b) The colored circles show the chromaticities of the eight reflectances under equal energy white. The plus symbol shows the chromaticity of the flat reflectance under equal energy white. The black dashed line indicates the black-body locus. [(c)–(f)] Effects of environmental illumination on the mean chromaticity of rendered objects for the conditions in Experiment 1. The square and triangle symbols indicate sphere and bumpy conditions, respectively. The semi-transparent symbols are re-plotted from panel (b), for comparison purposes. (c) Matte objects under Environment 1. (d) Matte objects under Environment 2. (e) Glossy objects under Environment 1. (f) Glossy objects under Environment 2.

Measuring thresholds of reflectance discrimination required continuous and systematic control of the spectral reflectance of the surface. Thus, we selected eight reflectance functions as shown in Fig. 3(a)

from a database of natural objects [48–50] and, by combining a spectrally flat (equal energy) reflectance with each of the eight reflectances in differing proportions, we controlled the reflectance along eight color directions. Reflectance functions were specified between 400 nm and 700 nm in 10 nm steps. In Fig. 3(a), each reflectance is normalized by its highest value for the sake of visibility; however, in the actual experiment, all reflectances were normalized to have equal luminance under an equal energy white illuminant. The colored circles in Fig. 3(b) show the chromaticities of the eight reflectances when rendered under equal energy white. The plus symbol is the chromaticity of the spectrally flat reflectance under equal energy white.

Figures 3(c)–3(f) show the effects of environmental illuminations on the mean chromaticity of objects for all conditions in Experiment 1. The semi-transparent symbols show the data points re-plotted from Fig. 3(b) for the purpose of comparison. We see that Environment 1 only minimally distorts the arrangement of eight chromaticities, whereas Environment 2 vertically expands the chromatic circle and shifts the overall position toward higher S/(L+M) and lower L/(L+M). The effect of shape on mean chromaticity is small, but the bumpy shape has systematically slightly lower S/(L+M), because the bumpy object has attached shadows that block some of the light coming from above (i.e., blue sky in this environment map). For glossy objects, however, the change in reflectance has less influence on the mean chromaticity. We independently rendered matte objects and glossy objects, and scaled the corresponding matte and glossy images to keep the total energy in the environmental illumination constant for both objects. Consequently, when more light is reflected in the specular component, less is available for the diffuse component, so the surface spectral reflectance has less influence on the proximal image.

### C. Observers and Ethical Approval

Three observers (JH, SR, and TM; TM is the first author of the study) participated in Experiment 1. For Experiment 2, JH and TM were again recruited along with two additional observers (AKH, TD). All observers were aged 23 to 26 and had corrected visual acuity and normal color vision as assessed by Ishihara pseudo-isochromatic plates. This study was approved by the Medical Sciences Inter-Divisional Research Ethics Committee at the University of Oxford, in agreement with the Declaration of Helsinki.

### D. Procedure

**Fig. 4.**
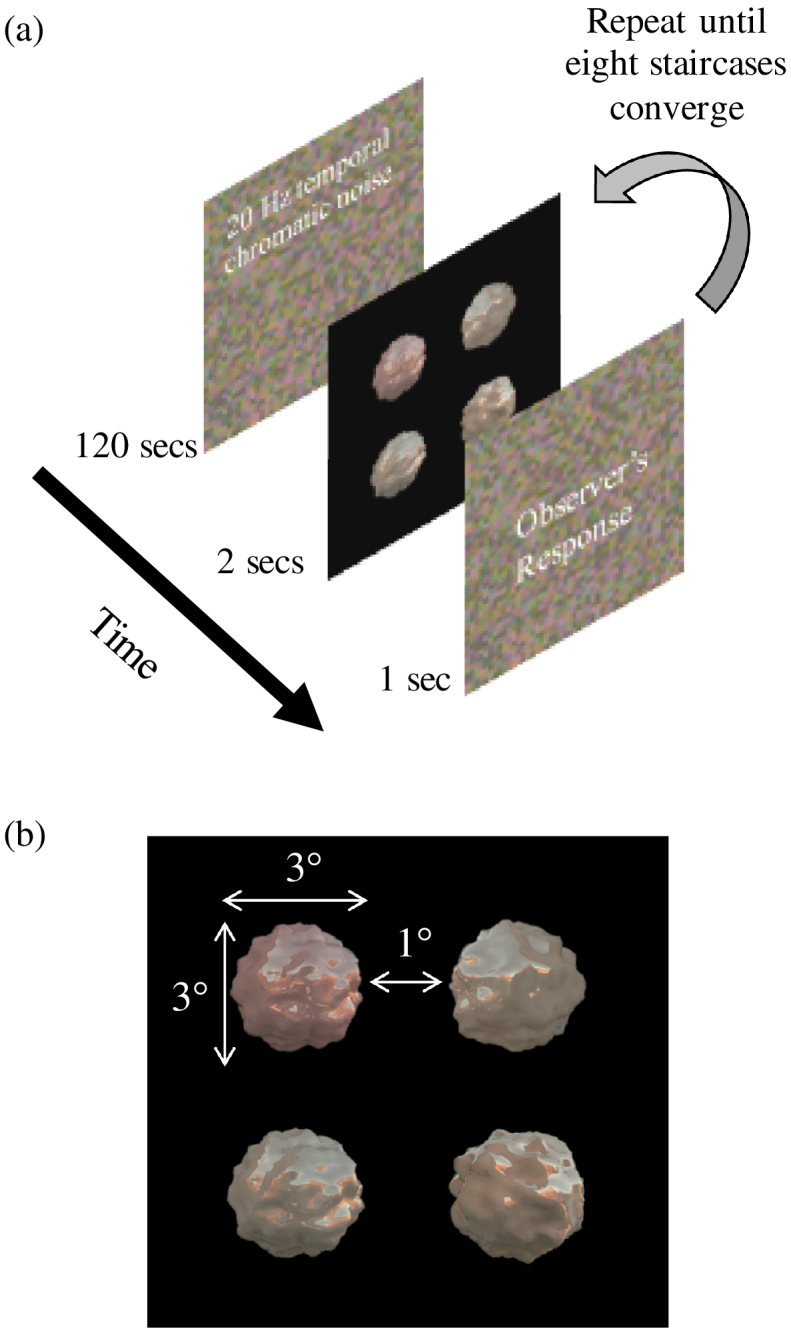
Schematic illustration of the procedure. (a) After an initial adaptation to chromatic noise, a series of 4AFC trials was presented, until eight interleaved staircases converged. See the main text for details. (b) An example of stimulus presentation on a single trial. Four objects were simultaneously presented for 2 s. The observer’s task was to select one with a different spectral reflectance. All objects in a trial had the same level of specularity (either matte or glossy) and the same 3D shape (either sphere or bumpy). The viewpoint from which the objects were rendered was different for the four objects presented, and so the distractors were not identical to one another.

Before each block, observers adapted to random-dot 20 Hz temporal chromatic noise for 2 min [Fig. 4(a)

]. The noise consisted of the chromaticities of the eight reflectances under equal energy white with equal probability.

Thresholds were then measured using an adaptive staircase method based on the procedure implemented by the Palamedes toolbox [51]. During each trial, four objects were simultaneously presented for 2 s, as shown in Fig. 4(b). The four objects were always presented from a different camera angle in order to prevent the observers from comparing the colors of specific points across objects. We prepared 36 viewpoints (from 0° to 350° with 10° steps) for each rendered image. On each trial, the viewpoint for each object was assigned randomly. Three distractor objects always had a flat reflectance, whereas the other object, the target, had a reflectance that was biased toward one of the eight hue directions defined by the eight reflectances. The observers’ task was to indicate the object that had a different reflectance. The observers were instructed to find the object with a different surface color. There was no fixation point, and the observers were instructed to move their eyes to look at each object. A participant’s response determined how the surface reflectance of the target object was updated for the next trial: when the response was incorrect, the update was toward the target reflectance; when the response was correct, the update was toward the flat reflectance. There was a 1-s inter-trial interval filled with the same temporal chromatic noise used at the start of the block. Thresholds for the eight hue directions were measured in parallel in the same block (eight interleaved staircases). The presentation order of the eight hues was randomized. The staircases were judged to have converged when the standard deviation of the stimulus magnitude of the last 10 trials was smaller than 2% of the prepared stimulus range. This was a conservative criterion as the standard deviation of the thresholds was generally larger. In addition, trajectories of all staircases were manually checked by the experimenter. Each staircase typically needed 20–30 trials to converge. When one staircase reached the threshold, it dropped out; trials continued until all eight staircases converged.

One session consisted of eight blocks (conditions) in Experiment 1 and four blocks in Experiment 2. Specific conditions are detailed in each experimental section. Observers performed five sessions in total for each experiment. All procedures, including the observers’ task, were identical for both experiments.

## 3. EXPERIMENT 1

Experiment 1 was designed to explore the effects of environmental illumination, specularity, and shape of objects on the thresholds of reflectance discrimination.

### A. Conditions

We used eight conditions, consisting of combinations of two environmental illuminations (Environment 1 and Environment 2), two specular levels (glossy, and matte as a control), and two shapes (sphere and bumpy). Table 1

**Table 1.** Summary of Conditions in Experiment 1 with Example Objects That Have Spectrally Flat Reflectance Functions

Illumination		Environment 1		Environment 2	
Shape		Sphere	Bumpy	Sphere	Bumpy
Specularity	Matte	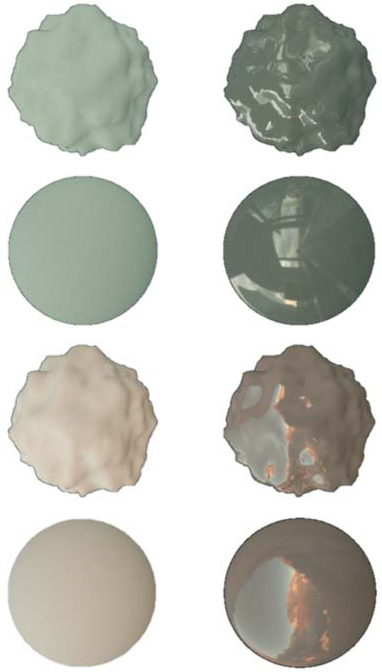			
	Glossy				

shows example objects with a spectrally flat reflectance rendered under settings for all eight conditions.

### B. Results and Discussion

Figure 5(a)

**Fig. 5.**
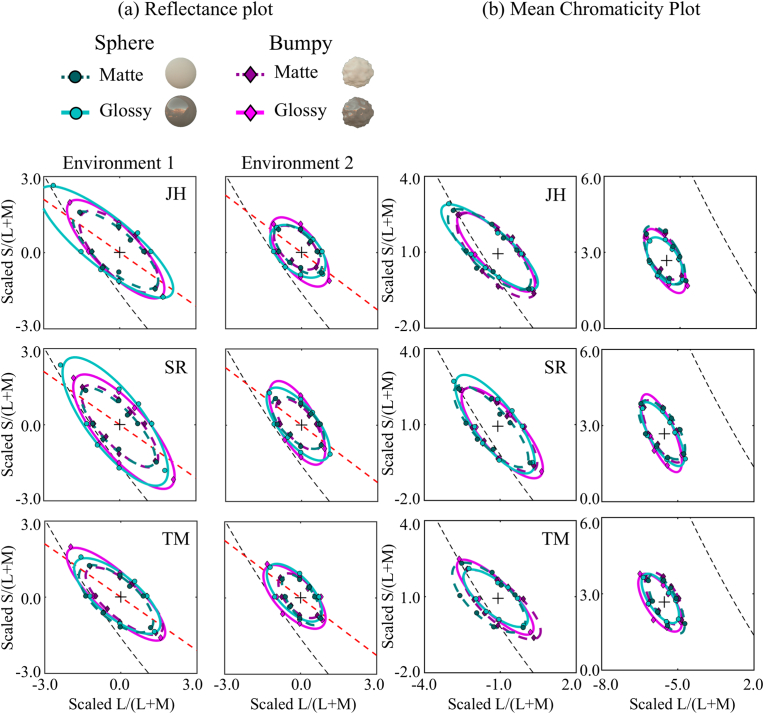
Results from Experiment 1. (a) Reflectance discrimination thresholds plotted on a reflectance-based plot, where each data point is represented by the chromaticity of the reflectance function at threshold, viewed under equal energy white. (b) Reflectance discrimination thresholds plotted on a mean-chromaticity-based plot, where each data point is represented by the mean chromaticity of the object at threshold. (a), (b) Different rows show data from different observers. Different columns show different environmental illuminations. The black dashed lines indicate the black-body locus. The red dashed lines in panel (a) indicate the axis that exhibits the maximum variation in chromaticity of the environmental illumination. Data are plotted in a scaled MB chromaticity diagram, where equal energy white corresponds to the origin and each axis is independently scaled by chromatic discrimination thresholds along L/(L+M) and S/(L+M) that were measured prior to the experiment.

shows thresholds of reflectance discrimination for all observers. The data were averaged across five repetitions. Each point indicates the chromaticity of the threshold reflectance under equal energy white. This reflectance-based plot is not the only way to represent results, but it allows us to compare the results directly across conditions; i.e., overlap of the plots means that same physical reflectance was chosen as a threshold. Data are plotted in a scaled MacLeod–Boynton (MB) chromaticity diagram, where equal energy white corresponds to the origin and each axis is scaled independently by chromatic discrimination thresholds along L/(L+M) and S/(L+M) measured prior to the experiment. The left column shows the results for Environment 1, whereas the right column shows the results for Environment 2. In the figures, the cyan circle symbols represent the sphere condition, whereas the magenta diamond symbols represent the bumpy condition. The dashed and solid lines are the matte and glossy conditions, respectively. The black dashed line is the black-body locus. The red dashed line is the axis that corresponds to the maximum variance of chromaticity in the environment map.

Three notable results emerge from Environment 1. First, the glossy condition shows a slightly larger discrimination ellipse than the matte condition. Second, the effect of shape is small. Third, and importantly, the discrimination ellipse is tilted along the direction of maximum variability of colors in the environmental illumination (red dashed line). These trends were consistent across all observers.

For the results in Environment 2, we see generally similar trends to Environment 1, but the overall ellipses are noticeably smaller. One of the differences between the two environments is that chromaticity does not distribute around equal energy white in Environment 2; this might help observers to separate the colors that belong to the illumination and those that belong to the surface.

These results are presented from the perspective of reflectance. However, the actual mean chromaticities of the rendered objects under each environment are shifted from these points, as shown in Figs. 3(c)–3(f). Thus, Fig. 5(b) provides another representation where each point indicates the mean chromaticity of objects with the threshold reflectance. Strictly speaking, mean chromaticity changes depending on the camera angles presented, but the effects of viewpoint were averaged.

A simple discrimination model based on mean chromaticity, which assumes that observers are able to make a discrimination when the mean chromaticity of the target object becomes far enough from the mean chromaticity of distractor objects, would predict circular discrimination ellipses. However, we see that plotting thresholds in such a way does not eliminate the elongation of the ellipse or the way it is tuned in color space. Consequently, the analysis of chromatic thresholds confirms that we have worse discrimination in a direction that corresponds to the major axis of variation of colors in the environment. A plausible account would say that spatial color variation of the specular component is greater in that direction and selectively masks co-aligned color differences [39] that arise from reflectance changes.

**Fig. 6.**
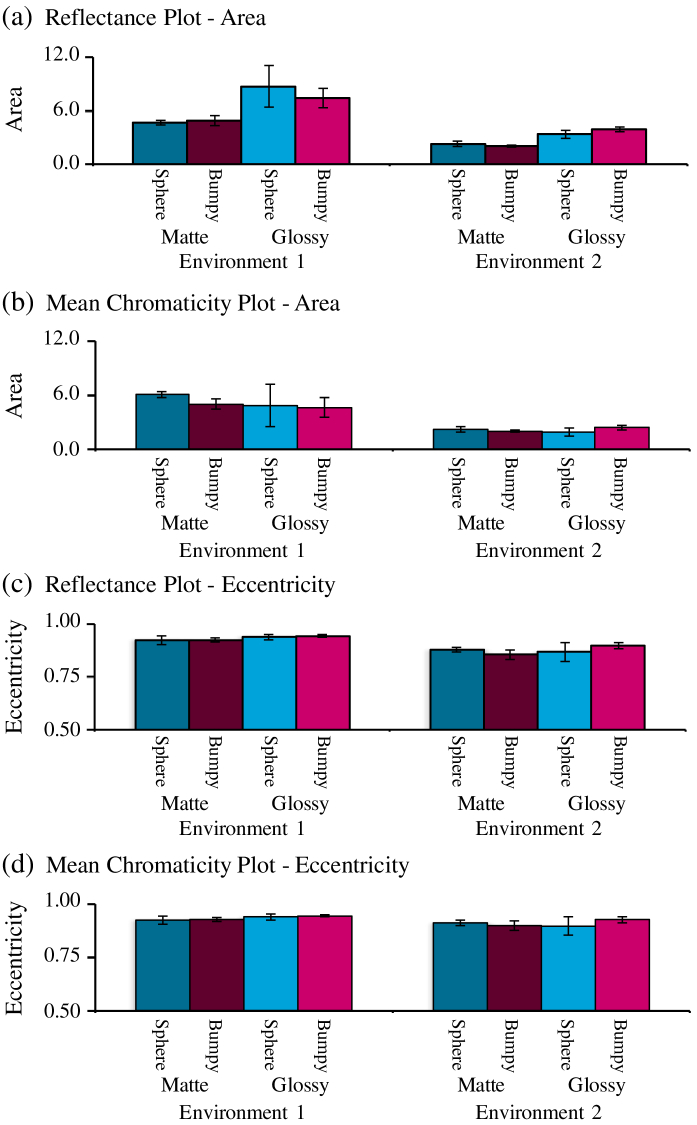
Summary of reflectance discrimination performance across conditions of Experiment 1. (a) Mean area of ellipses measured on the reflectance-based plot. (b) Mean area of ellipses measured on the mean-chromaticity-based plot. (c) Mean eccentricity of ellipses measured on the reflectance-based plot. (d) Mean eccentricity of ellipses measured on the mean-chromaticity-based plot. Error bars indicate ±1 S.E. across all observers.

The enclosed area and the eccentricity of the discrimination ellipses provide summary measures of discrimination performance. Figure 6

allows us to compare the area and eccentricity for all conditions in terms of the reflectance plot [Figs. 6(a) and 6(c)] and the mean-chromaticity plot [Figs. 6(b) and 6(d)]. Error bars indicate ±1 S.E. across observers. From the area plot [Fig. 6(a)], we see that discrimination is consistently worse in Environment 1. Plotting in terms of mean chromaticity [Fig. 6(b)] rather than reflectance reduces the differences between matte and glossy conditions. The eccentricities of the ellipses are nearly equal across conditions [Figs. 6(c) and 6(d)] meaning that the ratio between major and minor axes remained constant.

A three-way repeated-measures analysis of variance (ANOVA) was performed using environmental illumination (Environment 1 and Environment 2), specularity (matte and glossy), and shape of object (sphere and bumpy) as within-subject factors for the area of ellipses in the reflectance plot. We found a significant main effect of environmental illumination [F(1,2)=48.8, p=0.0199], whereas the main effects of specularity and of shape were not significant [F(1,2)=17.9, p=0.0516; F(1,2)=0.58, p=0.526, respectively]. Also, no significant interactions were observed [environmental illumination×specularity, F(1,2)=3.46, p=0.204; specularity×shape, F(1,2)=0.29, p=0.644; environmental illumination×shape, F(1,2)=2.82, p=0.235; environmental illumination×specularity×shape, F(1,2)=2.20, p=0.276].

In the same way, a three-way repeated-measures ANOVA was performed for the averaged area of ellipses for the mean chromaticity plot. We again found a significant main effect of environmental illumination [F(1,2)=66.3, p=0.0148], whereas the main effects of the specularity and shape of objects were not significant [F(1,2)=1.2, p=0.388; F(1,2)=2.1, p=0.284, respectively]. Again, no significant interactions were observed [environmental illumination×specularity, F(1,2)=1.69, p=0.323; specularity×shape, F(1,2)=5.35, p=0.147; environmental illumination×shape, F(1,2)=6.93, p=0.119; environmental illumination×specularity×shape, F(1,2)=0.01, p=0.929].

For the eccentricity measure, a three-way repeated-measures ANOVA showed the following results: in terms of the reflectance plot [Fig. 6(c)], we found a significant main effect of specularity [F(1,2)=31.7, p=0.0301], whereas the main effects of environmental illumination and shape were not significant [F(1,2)=13.7, p=0.0659; F(1,2)=7.81, p=0.108, respectively]. Also, the interaction between the three factors was not significant [environmental illumination×specularity, F(1,2)=0.001, p=0.978; specularity×shape, F(1,2)=2.49, p=0.255; environmental illumination×shape, F(1,2)=0.001, p=0.978; environmental illumination×specularity×shape, F(1,2)=1.96, p=0.296]. In terms of the mean-chromaticity plot [Fig. 6(d)], we found no significant main effect of environmental illumination, specularity or shape [F(1,2)=2.68, p=0.243; F(1,2)=10.7, p=0.0821; F(1,2)=3.39, p=0.207, respectively]. Also, no significant interaction was observed [environmental illumination×specularity, F(1,2)=0.28, p=0.650; specularity×shape, F(1,2)=1.09, p=0.406; environmental illumination×shape, F(1,2)=0.03, p=0.878; environmental illumination×specularity×shape, F(1,2)=2.29, p=0.269].

Therefore, the environment in which objects exist affects our performance of reflectance discrimination, whereas the properties of the objects (shape and specularity) have little impact.

One of the interesting effects we found in Experiment 1 was that discrimination ellipses were tuned in a certain way. It is known that the chromaticities of lights available in natural environments are typically distributed along the black-body (or daylight) locus [52]. If this is the case, then we would likely have a hue-dependent discrimination ability in general. It would thus be useful to determine whether the observed elongated ellipse is due to the specific environments under which stimuli were rendered or if it is due to the variation in natural environments that we experience in daily life. In other words, it is possible that sensitivity along the black-body locus is reduced because we adapt to such a chromatic distribution in daily life. Such an effect of long-term adaptation to environmental stimuli has been reported in past studies of color appearance [53–56], and it may be possible that a similar effect is found for threshold-based measurements. If that is the case, then we should observe this tuning effect with whatever scene we employ in a rendering process. To address this question, we decided to invert the chromatic distribution of Environment 1 to change the direction of chromatic variation as shown in Fig. 2(c), and conduct Experiment 2 using the same procedures as for Experiment 1. There is no unique way to invert the chromatic distribution of environmental illumination, but we chose to first convert RGB values to LMS values, and then to reflect the chromaticities in a line parallel to the L/(L+M) axis of MB space, where the inversion axis intersected the L/(L+M) value of equal energy white. Colors that were out of gamut as a result of this inversion were mapped to the gamut boundary (a manipulation known as “clipping”). Finally, the reverse transformation was performed to return the MB coordinates to RGB values for rendering and display.

## 4. EXPERIMENT 2

### A. Conditions

In Experiment 2, there were four conditions: a factorial combination of two environmental illuminations (Environment 1 and the inverted environment) and two specular levels (matte, and glossy). In this experiment, we used only bumpy stimuli.

### B. Results and Discussion

Figure 7

**Fig. 7.**
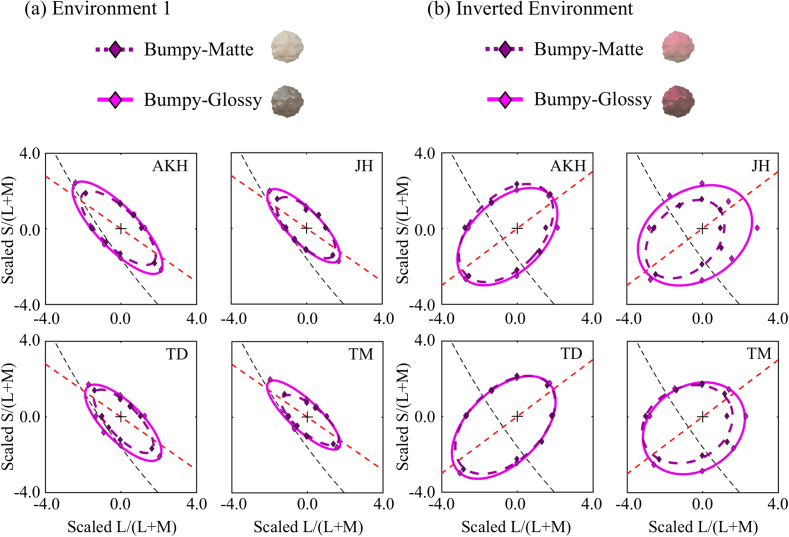
Results from Experiment 2, with reflectance discrimination thresholds plotted on a reflectance-based plot [analogous to Fig. 5(a)]. (a) Data obtained with stimuli rendered under Environment 1. (b) Data obtained with stimuli rendered under the chromatically inverted version of Environment 1. The axis scaling is the same as in Fig. 5.

shows the results for Experiment 2. We see that inverting the chromatic distribution of the environment also inverts the discrimination ellipse. Therefore, the tuned ellipses observed in Experiment 1 were influenced by the environment in which the objects were rendered rather than being influenced solely by the natural environment in which we live. Also, we found that under the atypical lighting environment, the discrimination ellipses became less elongated, and larger. Figure 8

**Fig. 8.**
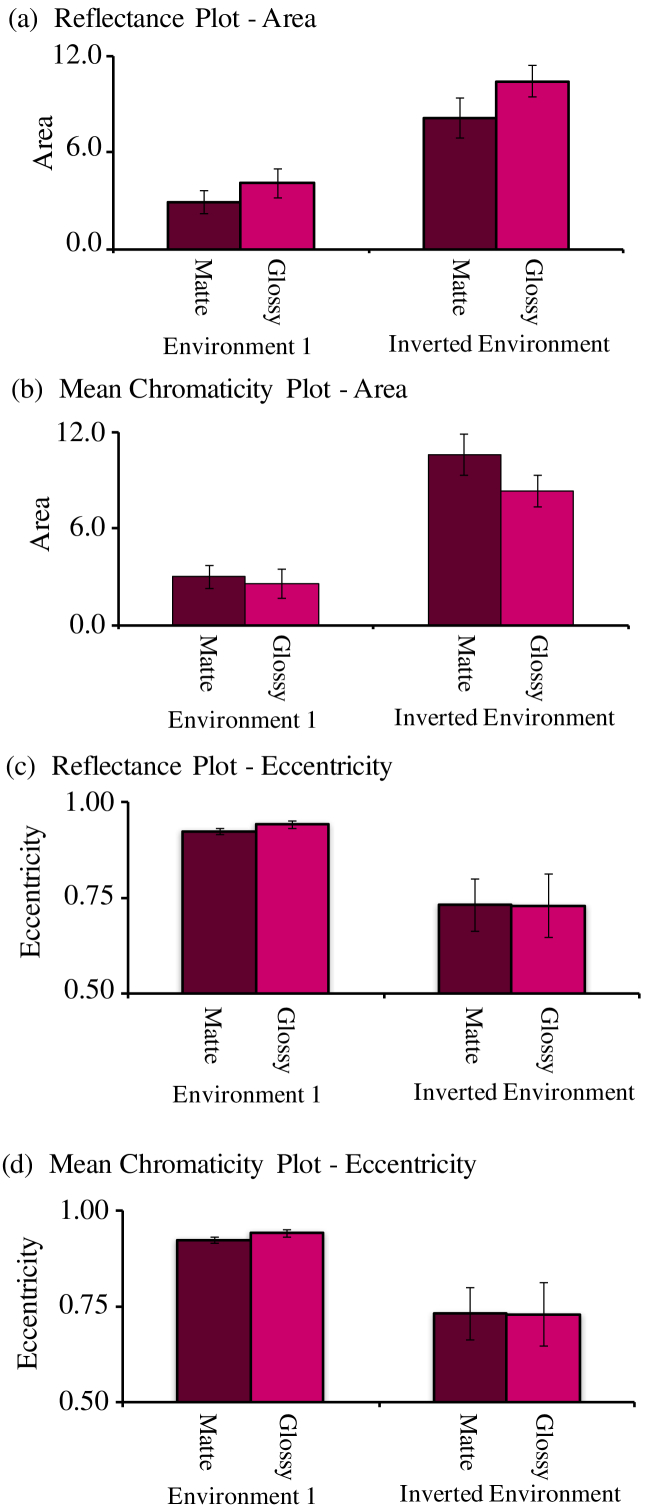
Summary of reflectance discrimination performance across conditions of Experiment 2. (a) Mean area of ellipses measured on the reflectance-based plot. (b) Mean area of ellipses measured on the mean-chromaticity-based plot. (c) Mean eccentricity of ellipses measured on the reflectance-based plot. (d) Mean eccentricity of ellipses measured on the mean-chromaticity-based plot. Error bars indicate ±1 S.E. across all observers.

compares the area and eccentricity of ellipses in both a reflectance-based plot [Figs. 8(a) and 8(c)] and a mean-chromaticity-based plot [Figs. 8(b) and 8(d)].

A two-way repeated-measures ANOVA was performed using environmental illumination (Environment 1 and inverted environment) and specularity (matte and glossy) as within-subject factors separately for each panel in Fig. 8. For the area measure, the analysis produced the following results: in terms of the reflectance plot [Fig. 8(a)], we found a significant main effect of environmental illumination [F(1,3)=168.9, p<0.001] and specularity [F(1,3)=10.6, p=0.0473]. The interaction between the two factors was, however, not significant. In terms of the mean-chromaticity plot [Fig. 8(b)], we found a significant main effect of environmental illumination [F(1,3)=358.2, p<0.001], whereas the main effect of specularity was not significant [F(1,3)=3.7, p=0.150]. Again, no significant interaction was observed. For the eccentricity measure, the analysis produced the following results: in terms of the reflectance plot [Fig. 8(c)], we found a significant main effect of environmental illumination [F(1,3)=19.8, p=0.0211], whereas the main effect of specularity was not significant [F(1,3)=3.06, p=0.179]. The interaction between the two factors was not significant. In terms of the mean-chromaticity plot [Fig. 8(d)], we found a significant main effect of environmental illumination [F(1,3)=80.3, p=0.00293] and of specularity [F(1,3)=10.7, p=0.0467]. Again, no significant interaction was observed.

The data from Experiment 2 suggest that discrimination of spectral reflectances would not work well when the chromatic distribution of illumination does not follow the way in which colors distribute in natural environments, i.e., along the black-body locus. This in turn might imply that our visual system can exploit the statistical regularity of chromaticities in environmental illumination to separate which color variations across an object’s surface are likely to arise from the illumination conditions.

## 5. GENERAL DISCUSSION

### A. Overview

The purpose of the present study was to measure thresholds of reflectance discrimination for glossy and matte objects under various environmental illuminations. For matte objects, environmental variation in incident illumination expands the gamut of chromaticities contained in the diffuse component of the proximal image of the object [Fig. 1(b)]. For glossy objects, in addition to this variation in the diffuse component, the full variation of chromaticities in environmental illumination is carried unmodified in the specular component [Fig. 1(c)].

For a perfectly color-constant observer, the type of illumination should not affect the thresholds for reflectance discrimination. However, the results of Experiment 1 (Figs. 5 and 6) indicated that reflectance discrimination is actually not constant across environmental illuminations, suggesting that our ability to perceive surface color depends on the lighting environment in which objects are placed. It was also evident that discrimination ellipses were elongated along the direction of maximum variance of chromaticities in the environmental illumination map. The results of Experiment 2 (Figs. 7 and 8), where we used a chromatically inverted environmental illumination map, showed that this tuning effect seems to be dominated by the chromatic distribution of the environment used for rendering rather than a statistical invariance of natural environments. The chromatically inverted environment additionally gave rise to larger discrimination ellipses than the typical environment. These findings were consistent across all observers.

### B. Available Cues

The 4AFC procedure for reflectance discrimination is a relative judgment that does not necessarily require estimating surface or illuminant properties. Instead, global statistics, such as mean chromaticity, may have allowed observers to select the odd-one-out. It is possible therefore to consider the task as one of color discrimination in the presence of a chromatic noise mask, where the noise arises from the spatial variation in incident illumination. For matte objects, which contain only a diffuse image component, the spatial chromatic noise is low-contrast and low-spatial-frequency; however, for glossy objects, which additionally include a specular image component, the spatial chromatic noise is high-contrast and contains variation at many spatial frequencies, including sharp edges. There are many studies on color discrimination with chromatic noise [39,42,57]. They essentially show that adding chromatic noise elongates the discrimination ellipse along the direction in which the chromaticity of the noise extends. This is consistent with the obliquely tuned discrimination ellipses we measure: for different illumination environments, the discrimination ellipse reliably aligns with the axis of maximum chromatic variation.

One limitation of our study was that stimuli were rendered from a single viewpoint and therefore presented without binocular disparity. Since diffuse and specular components differ in their imaging geometry, it is possible that disparity information would have helped observers parse the image and judge surface color. With similar rendered objects under simple illumination, motion parallax does not improve constancy [58]. However, depth information may be particularly important in complex lighting environments.

### C. Matte versus Glossy Objects

The chromatic variation across the image of the object is much greater for glossy objects than for matte objects. Yet observers’ abilities to discriminate spectral reflectances were not significantly different for the two types of object (no main effect of specularity for the ANOVA in Experiment 1 or Experiment 2). The small, consistent, trend observed in both Experiment 1 and Experiment 2 toward poorer discrimination performance for glossy objects when analyzed in terms of reflectance was eliminated or reversed when discrimination ellipses were plotted with respect to mean chromaticity. This relative improvement for glossy objects when plotted in chromaticity space is consistent with the stimulus properties: when more light is reflected in the specular component, less is available for the diffuse component, so a given surface spectral reflectance has less influence on chromaticities in the proximal image. However, the overall similarity of performance with matte and glossy objects is somewhat counterintuitive.

As noted above, glossy objects exhibit much greater variation in chromaticity across their surface. Thus, previous measurements of color discrimination in noise [39] would predict higher thresholds for reflectance discrimination of glossy objects. This raises the possibility that, unlike random chromatic noise, specular reflections may contain some regularities specific to the illumination, which may help the visual system to eliminate the influence of specular reflection effectively. These regularities may be in the chromatic or spatial properties of the noise, and we discuss each possibility below.

### D. Chromatically Typical and Atypical Lighting Environments

In addition to the finding that discrimination ellipses align to the major axis of chromatic variation in the environmental illumination used for rendering, our second major result was that discrimination abilities became worse under the atypical environmental illumination in Experiment 2. This decline in performance was accompanied by a reduction in the eccentricity of the ellipse. Had discrimination thresholds depended only on the chromatic distribution of the environmental illumination used for rendering, there would have been no change in the overall area of the ellipses between the typical and chromatically inverted environments, and no change in the eccentricity of the discrimination ellipses. One explanation would be that, irrespective of the particular environment used for rendering, chromatic change parallel to the black-body locus is likely to be assigned to the illumination (and not to a surface reflectance) because chromaticities of lights in natural environments are distributed along the black-body locus [52,59]. This is consistent with the idea of using priors such as statistical regularities in natural environments to solve the problem of color constancy [60,61].

The results of Experiment 2 raise the question of whether the visual system understands the chromatic properties of specular reflection of incident illumination. Further investigation is needed to address this question, but it is possible that the visual system internalizes the chromatic regularities in environmental illumination to separate the diffuse and specular components of the proximal image of an object. Indeed, several studies have reported the importance of the black-body (or daylight) locus for color vision [40,62]. The chromatic tuning of natural environmental illumination is heavily determined by the chromatic tuning of natural illuminants. Conversely, for object colors, we know that the spectral reflectances of natural objects are less constrained than the spectral content of natural illuminants. However, from theoretical arguments, distributions of spectral reflectances rendered under equal energy white are expected to show a negative correlation in L/M-opponent and S-opponent signals [62,63]. We conducted our own analysis and confirmed that the chromaticity of 4824 surface spectral reflectances of natural objects [48–50] under equal energy white light is distributed broadly along the black-body locus. Thus, it seems unlikely that color changes along the black-body locus could be strongly diagnostic of the difference between reflectance-based or illuminant-based variation in the proximal image. Interestingly, chromaticities in #theDress, which is an “illusion” that gives rise to observer-dependent color appearance, are also spread along the black-body locus [64,65], and this may explain why observers have difficulties in separating surface and illuminant information in this image. The asymmetric effects of chromatic change along particular directions in the color space would be an interesting topic to pursue further.

### E. Spatial Signatures of Lighting and Reflectance Changes

The reflectance discrimination paradigm used here has parallels with the paradigm of illuminant discrimination [40,41]. The stimuli for reflectance discrimination offer a single, spatially uniform, surface spectral reflectance, with complex spatial variation in incident illumination; the stimuli for illuminant discrimination offer complex spatial variation in reflectance, with uniform illumination. Both reflectance discrimination and illuminant discrimination seem to be poor along the black-body or daylight locus. An important link between the two tasks is that they may both depend in part on the discrimination of mean chromaticity (or low-spatial-frequency chromatic signals), which raises the possibility that the chromatic tuning effects are specific to spatial scale.

In many studies, it has been claimed that the problem of color constancy is equivalent to a problem of illuminant estimation [1]. That is, once the visual system recognizes the illumination color, the color constancy can be readily implemented by globally subtracting the illuminant color from the whole field of the view. This idea has an attractive simplicity, and it may also be true under constrained situations in which objects are matte and a scene has only a single light source. However, the spatial variation in chromaticity and luminance that derives from the specular component in the image of a glossy object seems to challenge simple estimates of illuminant color because local illuminant cancellation is required [31], which seems to be unrealistic from the perspective of computational cost. Instead, what the visual system needs is a way to extract the component that attaches to the surface property of spectral reflectance.

When the object is a smooth sphere, the specular reflection preserves the spatial structure of the surrounding environment, as shown in Fig. 1(a). Thus, one might expect an advantage for smooth versus bumpy spheres, since smooth spheres might allow the visual system to more readily understand how the specular components are distributed on the surface of an object. However, in the present study, when objects were presented in a dark void, there was no significant difference in thresholds between the sphere and bumpy conditions. This suggests that although the spatial patterning of specular reflections provides important information about the shape of objects [66], spatial variations that arise from surface geometry do not have a strong effect on the ability to judge surface colors. In a follow-up study, we intend to present rendered objects within the full rendered environment to assess whether contextual information helps with judgments of surface color.

The study of material perception, especially considering color-related effects, has started only recently. Such work is expected to provide new insights to understand the color that is produced by a complex interaction between illumination and surface properties. At the same time, the field of color constancy needs to move from simplified stimuli toward more complex stimuli that more fully reflect the properties of real objects and their lighting environments. This work represents a preliminary step and it will be important to extend the investigation to different surface properties, shapes, and lighting environments so as to assess the generalizability of the findings.
